# Cooperative Jammer Selection for Secrecy Improvement in Cognitive Internet of Things

**DOI:** 10.3390/s18124257

**Published:** 2018-12-04

**Authors:** Ping Xie, Ling Xing, Honghai Wu, Jung Taek Seo, Ilsun You

**Affiliations:** 1 Information Engineering College, Henan University of Science and Technology, Luoyang 471023, China; xieping_1984@bupt.edu.cn (P.X.); xingling_my@163.com (L.X.); whh1010@gmail.com (H.W.); 2 Department of Information Security Engineering, Soonchunhyang University, Asan-si 31538, Korea; seojt@sch.ac.kr

**Keywords:** aided opportunistic jamming, artificial noise, cognitive Internet of things, intercept probability, physical layer security

## Abstract

Smart homes can improve the quality of life and be implemented by Internet of Things (IoT) technologies. However, security is a very important issue in smart homes. For this reason, we propose a secrecy transmission protocol for primary user (PU) by selecting friendly jammer in cognitive IoT model. In particular, a secondary transmitter (ST) is selected to transmit secondary signals by the PU’s frequency spectrum, while another ST is chosen to transmit artificial noise to protect the transmission confidentiality of the PU against eavesdropping. Moreover, two selection schemes are presented to confirm the former and the latter ST, and the goal is to optimize the secondary transmission performance and the primary security performance, respectively. For the non-security model and the proposed protocol, we derive the closed-form expressions of the intercept probability and the outage probability for the PU. We also obtain the closed-form expression of outage probability for the secondary user. The numerical results show that the security performance of the PU is significantly enhanced in our protocol compared to the non-security model. In addition, the outage performance of the secondary users is also improved in high secondary transmit SNR region.

## 1. Introduction

The Internet of Things (IoT) is an emerging wireless application [1] and has many applications [2,3]. Many techniques for IoT have arisen in recent years such as adaptive monitoring techniques [4,5,6]. Moreover, IoT technologies can implement smart homes, which can improve the quality of life. However, the security and privacy problems are very important in smart homes [7] and have received significant interest [8,9,10,11,12]. Furthermore, combining cognitive radio technique and IoT, Cognitive Internet of Things (CIoT) is proposed, which is an enhanced IoT paradigm. However, the available bandwidth for IoT is very limited. Thus, the spectrum efficiency is a key issue for IoT design [13,14]. To improve the utilization efficiency of radio spectrum, Cognitive Radio (CR) [15] is a promising technology [16]. In Cognitive Radio Networks (CRN), unlicensed users opportunistically access to the licensed spectrum band [17]. Furthermore, unlicensed users cannot harm the performance of primary users. However, since the spectrum access is dynamic and the communication mode is broadcast communication in wireless communication, any unlicensed users and eavesdroppers can have access to the shared spectrum. Therefore, the eavesdroppers readily overhear any active transmissions over wireless networks. However, cognitive radio technology also introduces some new security threats, e.g., using the shared spectrum by selfish behavior, reporting false sensing information, etc. Therefore, ensuring security is a key issue in CIoT.

To ensure security, physical-layer security technology is an effective confidentially protection mechanism [18,19,20,21,22]. In Ref. [23], when the wiretap channel condition between a source node and an eavesdropper node is worse than the channel condition between the source node and the destination node, the source node can successfully communicate with the destination node in perfect secrecy. Ref. [22] emphasized that both the primary users (PUs) and secondary users (SUs) must be defended from eavesdropping in cognitive networks. Specifically, it is legitimate that the SUs are allowed to access the primary spectrum by cooperating with the PUs, where the SUs act as a relay or a friendly jammer to elevate the PU’s secrecy [24]. Some studies [25,26] reveal that resource allocation is an efficient approach to ensure the PU’s security requirement while achieving good transmission performance for the SUs who cooperate with the PUs. In addition, both the secure communications for PUs and SUs are considered in Ref. [25]. In contrast, Refs. [27,28,29] studied some transmission schemes to maximize secrecy rate or to minimize secrecy outage probability for the SUs in the underlay cognitive models, respectively. In addition, the user selection in cooperative transmission is also an efficient method to enhance the security performance for communication systems due to the multiuser gain. The security enhanced technologies of the SUs is investigated with the user selection in Refs. [21,30,31]. However, transmission protocols for improving the secrecy performance of the PUs are barely known. How to design the transmission protocol for protecting the PU’s security requirement remains a crucial issue in cognitive Internet of things model, where home terminal-to-terminal communication coexists with uplink or downlink of the femtocell station.

To improve the primary secrecy performance and secondary outage performance, we employ cooperative jammer and multi-user diversity technology in this paper. Namely, artificial noise is transmitted by selecting a secondary transmitter (ST), which can improve the outage performance of the primary system. Moreover, an ST has access to the primary spectrum if it can improve the outage performance of the secondary performance and satisfy the interference threshold. To encourage the secondary transmitter to act as a friendly jammer, the interference threshold for secondary system is relaxed by primary system in this paper. The main contributions are summarized as follows:

- We propose a ST cooperative transmission protocol by selecting jammer, which transmits an artificial noise to disturb the eavesdropper.
- We propose a selection scheme to determine the friendly jammer and secondary signal transmitter. The ST, which can provide the smallest intercept probability, is chosen as the friendly jammer to transmit artificial noise.
- We derive the closed-form expressions of the intercept probability and the outage probability for the primary system over Rayleigh fading channels, respectively. We also derive the outage probability of the secondary user over Rayleigh channels.

The remainder of the paper is organized as follows. The system model of cooperative jammer selection for primary systems is provided in Section 2. Section 3 analyzes the performances of transmission and security for our proposed protocol. Section 4 provides numerical simulations for the proposed protocol. Section 5 concludes this paper.

*Notations*: The channels coefficients over links PS→PD, PS→SR, PS→E, ${\mathrm{ST}}_{i}\to \mathrm{PD}$, ${\mathrm{ST}}_{i}\to \mathrm{SR}$, ${\mathrm{ST}}_{o}\to E$, and ${\mathrm{ST}}_{i}\to E$ are denoted by $h_{P}$, $h_{\mathrm{PS}}$, $h_{\mathrm{PE}}$, $h_{S_{i}P}$, $h_{S_{i}}$, $h_{S_{o}E}$, and $h_{S_{i}E}$, respectively. $R_{P}$ denotes the minimum rate of transmission for primary systems. We also use $R_{S}$ to denote the minimum rate of transmission for secondary systems. The transmit power of ST and PT are denoted by $P_{P}$ and $P_{S}$, respectively. The expectation of a variable *X* is denoted by E[X]. The probability of a variable *X* is denoted by Pr{X}.

## 2. The System Models and the Selection Schemes for STs

**Figure 1:**
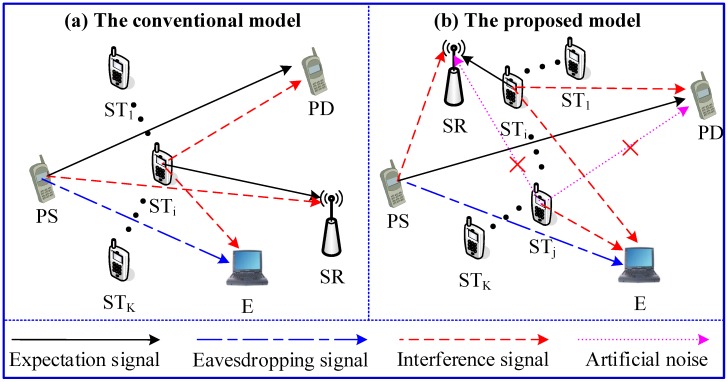
The system models.

In this section, we propose a ST cooperative transmission protocol and a selection scheme to determine the friendly jammer and secondary transmitter. Figure 1b shows The system configuration of our protocols. The system model comprises a primary pair (PS-PD), an eavesdropper (E), a secondary receiver (SR) and K secondary transmitters ${\mathrm{ST}}_{i}$, where i∈I, I={1,…,K}. In this transmission models, one secondary user is selected as a friendly jammer to interfere with eavesdropping at first, which is denoted by ${\mathrm{ST}}_{o}$, o∈I. The other one has access to the licensed spectrum if the secondary transmission cannot cause an outage over link (PS→PD), which is denoted by ${\mathrm{ST}}_{i}$, i∈I and i≠o. However, the transmitted information of primary users can be overheard by the eavesdropper. To prevent eavesdropping, ${\mathrm{ST}}_{o}$ transmits the artificial noise to interfere the eavesdropper. In the proposed model, PD and SR know the information of the artificial noise and the eavesdropper does not know the information. Therefore, PD and SR will not be affected by the artificial noise, which may disturb the eavesdropper. In the proposed protocol, on the one hand, ${\mathrm{ST}}_{o}$, which provides the most optimal security performance, is selected as a cooperative jammer. On the other hand, if the best outage performance of the secondary system is achieved by selecting a secondary user ${\mathrm{ST}}_{i}$, and the interference threshold of the primary system is satisfied for the secondary user ${\mathrm{ST}}_{i}$, then ${\mathrm{ST}}_{i}$ has access to the licensed spectrum. Furthermore, we study two criterions, which are used to select the cooperative jammer and secondary information transmitter, respectively. In addition, we assume that $h_{v}∼\mathrm{CN}\left(0,\sigma_{v}^{2}\right)$, where $v\in \left\{P,{\mathrm{PS}}_{i},\mathrm{PE},S_{i}P,S_{i},S_{o}E,S_{i}E\right\}$. We also assume that noises are Additive White Gaussian Noise (AWGN) with zero mean and variance $N_{0}$.

### 2.1. The System Model Based on the Security Enhancement Approach by Friendly Jammer Selection

To ensure the Quality of Service (QoS) of the primary system, the interference to primary users caused by secondary users must be less than a given threshold (i.e., the interference threshold is satisfied by secondary users). The secondary users have access to the licensed spectrum if they satisfy the above condition. A collection of the secondary transmitters is expressed as S, in which all STs can meet the interference threshold. The transmission process of the proposed protocol is illustrated as follows.

When S=Ø, the primary signals are transmitted by PS, the artificial noise is transmitted by ${\mathrm{ST}}_{o}$, but the secondary transmission is interrupted and SR does not work. PD can eliminate perfectly the artificial noise, which leads to a serious threat to the correct reception of the primary signals at E. Thus, the received signals at PD and E in this case are given by

(1) $$y_{P}^{S=Ø}\left(t\right)=\sqrt{P_{P}}h_{P}x_{P}\left(t\right)+n_{P}\left(t\right),$$

and

(2) $$y_{E}^{S=Ø}\left(t\right)=\sqrt{P_{P}}h_{\mathrm{PE}}x_{P}\left(t\right)+\sqrt{P_{S}}h_{S_{o}E}x_{n}\left(t\right)+n_{E}\left(t\right),$$

where $x_{P}\left(t\right)$ and $x_{n}\left(t\right)$ represent the primary signal and the artificial noise, respectively. Furthermore, normalizing, $E[|x_{P}\left(t\right){|}^{2}]=1$ and $E[|x_{n}\left(t\right){|}^{2}]=1$. $n_{P}\left(t\right)$ and $n_{E}\left(t\right)$ denote the noises at PS and E, respectively. Hence, the instantaneous capacities of the channel PS→PD and the channel PS→E are given by

(3) $$C_{P}^{S=Ø}=\log_{2}\left(1+P_{P}{\left|h_{P}\right|}^{2}/N_{0}\right)$$

and

(4) $$C_{E}^{S=Ø}=\log_{2}\left(1+\frac{P_{P}{\left|h_{\mathrm{PE}}\right|}^{2}}{P_{S}{\left|h_{S_{o}E}\right|}^{2}+N_{0}}\right).$$

When S≠Ø, denoting $S=S_{l}$ and having $S\in \left\{Ø\right\}\cup S_{l}$, the primary signals, secondary signals and artificial noise are transmitted by PS, ${\mathrm{ST}}_{i}$ and ${\mathrm{ST}}_{o}$, respectively, in the same spectrum band, where ${\mathrm{ST}}_{i}\in S_{l}$, ${\mathrm{ST}}_{o}\in S_{l}$ and $l=1,2,\ldots ,2^{K-1}-1$. In this case, mutual interferences are aroused between the primary and secondary users. The artificial noise is eliminated perfectly at PD and SR, but leads a serious threat to the correct reception of the primary signals at E. Thus, the received signals at PD, SR and E are given by

(5) $$y_{P}^{S=S_{l}}\left(t\right)=\sqrt{P_{P}}h_{P}x_{P}\left(t\right)+\sqrt{P_{S}}h_{S_{i}P}x_{S}\left(t\right)+n_{P}\left(t\right),$$

(6) $$y_{S}^{S=S_{l}}\left(t\right)=\sqrt{P_{S}}h_{S_{i}}x_{S}\left(t\right)+\sqrt{P_{P}}h_{\mathrm{PS}}x_{P}\left(t\right)+n_{S}\left(t\right),$$

and

(7) $$y_{E}^{S=S_{l}}\left(t\right)=\sqrt{P_{P}}h_{\mathrm{PE}}x_{P}\left(t\right)+\sqrt{P_{S}}h_{S_{o}E}x_{n}\left(t\right)+\sqrt{P_{S}}h_{S_{i}E}x_{S}\left(t\right)+n_{E}\left(t\right),$$

where $x_{S}\left(t\right)$ is the secondary signal and $n_{S}\left(t\right)$ denotes the noise at SR. Moreover, we assume that $E[|x_{S}\left(t\right){|}^{2}]=1$. Hence, the capacities of the channels PS→PD, ${\mathrm{ST}}_{i}\to \mathrm{SR}$, and PS→E are given by

(8) $$C_{P}^{S=S_{l}}=\log_{2}\left(1+\frac{P_{P}{\left|h_{P}\right|}^{2}}{P_{S}{\left|h_{S_{i}P}\right|}^{2}+N_{0}}\right),$$

(9) $$C_{S}^{S=S_{l}}\log_{2}\left(1+\frac{P_{S}{\left|h_{S_{i}}\right|}^{2}}{P_{P}{\left|h_{\mathrm{PS}}\right|}^{2}+N_{0}}\right),$$

and

(10) $$C_{E}^{S=S_{l}}=\log_{2}\left(1+\frac{P_{P}{\left|h_{\mathrm{PE}}\right|}^{2}}{P_{S}|h_{S_{o}E}{|}^{2}+P_{S}{\left|h_{S_{i}E}\right|}^{2}+N_{0}}\right),$$

where o,i∈I and i≠o. The number of elements in set $S_{l}$ is denoted by L−1. It is easy to know $S_{l}=\left\{i|C_{P}\geq R_{P},i\in I,i\neq o\right\}$, ${\bar{S}}_{l}=\left\{i|C_{P}<R_{P},i\in I,i\neq o\right\}$ and $S_{l}\cup {\bar{S}}_{l}=\left\{{\mathrm{ST}}_{i}|i\in I,i\neq o\right\}$. Hence, if $C_{P}>C_{E}$, then the physical-layer secrecy is obtained. If $C_{P}<C_{E}$, the secrecy intercept event happens. Its definition refers to [32]. Hence, in wireless systems, the physical-layer security is measured by its probability. Two selection criteria of ${\mathrm{ST}}_{o}$ and ${\mathrm{ST}}_{i}$ are described in detail in the next subsection.

### 2.2. The Selection Schemes for $ST_{o}$ and $ST_{i}$

In the multi-users underlay cognitive model, the primary security performance, and the primary and secondary transmission performances are the three most important indicators in system performance analysis. Moreover, the security performance of primary users can be improved effectively since a secondary user acts as a friendly jammer to interfere eavesdropping. By choosing a suitable user as the friendly jammer will further enhance the primary security performance. To optimize the primary physical-layer security performance, a secondary transmitter is selected to serve as a cooperative jammer, we use ${\mathrm{ST}}_{o}$ to denote the secondary transmitter, which can provide the most optimal security for the primary. Thus, the selection criteria of ${\mathrm{ST}}_{o}$ can be written as

(11) $$J=\arg\min_{j\in \{1,\ldots ,K\}}\mathrm{Pr}\left\{C_{P}^{S=Ø}<C_{E}^{S=Ø}\right\}=\arg\max_{j\in \{1,\ldots ,K\}}{\left|h_{S_{j}E}\right|}^{2},$$

where $C_{P}^{S=Ø}$ and $C_{E}^{S=Ø}$ are calculated by Equations (3) and (4), respectively. In addition, the secondary transmission performance is significantly improved by cooperative rewards that some primary spectrum is released or the value of interference threshold is relaxed for secondary transmission. However, different secondary transmitters have different transmission efficiencies. To maximize the secondary transmission performance, a secondary transmitter is denoted by ${\mathrm{ST}}_{i}$ that satisfies the interference threshold. Moreover, ${\mathrm{ST}}_{i}$ has access to the licensed spectrum if the optimal outage performance of the secondary system is obtained by ${\mathrm{ST}}_{i}$. The selection criteria for ${\mathrm{ST}}_{i}$ can be written as

(12) $${\mathrm{ST}}_{i}=\arg\min_{{\mathrm{ST}}_{i}\in S_{l}}\mathrm{Pr}\left\{C_{S}^{S=S_{l}}<R_{S}\right\}=\arg\max_{{\mathrm{ST}}_{i}\in S_{l}}C_{S}^{S=S_{l}},$$

where $C_{S}^{S=S_{l}}$ is calculated by Equation (9). Therefore, we focus on the selection of the secondary, which can have access to the primary spectrum and can be the cooperative jammer.

### 2.3. The Conventional Non-Security Model

As shown in Figure 1a, the system model of the conventional non-security management protocol comprise of a primary pair (PS-PD), an eavesdropper (E), a secondary receiver (SR) and *K* secondary transmitters ${\mathrm{ST}}_{i}$ (1,…,K). This conventional model is a typical cognitive underlay system, where STs can have access to the primary spectrum and need to satisfy the interference threshold settled by primary system. Compared with the conventional model, we can see that the received signals at PD and SR and the corresponding instantaneous capacities are identical. In contrast, the received signals at E and the corresponding instantaneous capacities are different. Thus, if $S^{C}=Ø$, the received signals at E and the corresponding instantaneous capacities are given by

(13) $$y_{E}^{C}\left(t\right)=\sqrt{P_{P}}h_{\mathrm{PE}}x_{P}\left(t\right)+n_{E}\left(t\right)$$

and

(14) $$C_{E}^{C}\left(t\right)=\log_{2}\left(1+P_{P}{\left|h_{\mathrm{PE}}\right|}^{2}/N_{0}\right).$$

If the secondary signal is transmitted over primary spectrum (namely, $S^{C}=Ø$), the received signals at E and the corresponding instantaneous capacities are given by

(15) $$y_{E}^{C}\left(t\right)=\sqrt{P_{P}}h_{\mathrm{PE}}x_{P}\left(t\right)+\sqrt{P_{S}}h_{S_{i}E}x_{S}\left(t\right)+n_{E}\left(t\right)$$

and

(16) $$C_{E}^{C}=\log_{2}\left(1+\frac{P_{P}{\left|h_{\mathrm{PE}}\right|}^{2}}{P_{S}{\left|h_{S_{i}E}\right|}^{2}+N_{0}}\right).$$

## 3. Performance Analysis

### 3.1. The Primary Outage Probability for the Proposed Protocols

We use $\Omega_{P}$ to denote an event, which represents an occurrence of outage of the channel PS→PD. Hence, if $C_{P}^{S=Ø}<R_{P}$ or $C_{P}^{S\neq Ø}<R_{P}$, the event $\Omega_{P}$ occurs. Obviously, the secondary transmission may make the event happen when S≠Ø. Thus, we obtain

(17) $$P_{\mathrm{out}}=\mathrm{Pr}\left\{S=Ø\right\}\mathrm{Pr}\left\{\Omega_{P}|S=Ø\right\}+\sum_{l=1}^{2^{K-1}-1}\mathrm{Pr}\left\{S=S_{l}\right\}\mathrm{Pr}\left\{\Omega_{P}|S=S_{l}\right\}$$

and

(18) $$\mathrm{Pr}\left\{S=Ø\right\}=\prod_{i=1,i\neq o}^{K}\mathrm{Pr}\left\{C_{P}<R_{P}\right\}=\prod_{i=1,i\neq o}^{K}\mathrm{Pr}\left\{\frac{P_{P}{\left|h_{P}\right|}^{2}}{P_{S}{\left|h_{S_{i}P}\right|}^{2}+N_{0}}<2^{R_{P}}-1\right\},$$

where $C_{P}^{S=Ø}$ is given by Equation (3). Since $|h_{P}{|}^{2}$ and $|h_{S_{i}P}{|}^{2}$ are i.i.d. exponential distribution with parameters $1/\sigma_{P}^{2}$ and $1/\sigma_{S_{i}P}^{2}$, respectively, letting $X_{1}={\left|h_{P}\right|}^{2}$ and $X_{2}={\left|h_{S_{i}P}\right|}^{2}$, Equation (18) can be rewritten as

(19) $$\begin{array}{cc} \mathrm{Pr}\left\{S=Ø\right\} & =\prod_{i=1,i\neq o}^{K}\mathrm{Pr}\left\{C_{P}<R_{P}\right\}=\prod_{i=1,i\neq o}^{K}\mathrm{Pr}\left\{\frac{P_{P}X_{1}}{P_{S}X_{2}+N_{0}}\right\}\\ & =\prod_{i=1,i\neq o}^{K}\int_{0}^{\infty }\frac{1}{\sigma_{S_{i}P}^{2}}e^{-\frac{x_{2}}{\sigma_{S_{i}P}^{2}}}\int_{0}^{\rho_{P}\left(P_{S}x_{2}+N_{0}\right)/P_{P}}\frac{1}{\sigma_{P}^{2}}e^{\frac{x_{1}}{\sigma_{P}^{2}}}dx_{1}dx_{2}\\ & =\prod_{i=1,i\neq o}^{K}\left(1-\frac{\sigma_{P}^{2}P_{P}e^{-\frac{\rho_{P}N_{0}}{P_{P}\sigma_{P}^{2}}}}{\sigma_{P}^{2}P_{P}+\rho_{P}P_{S}\sigma_{S_{i}P}^{2}}\right). \end{array}$$

Furthermore, $\mathrm{Pr}\{\Omega_{P}|S=Ø\}$ and $\mathrm{Pr}\{S=S_{l}\}$ can be calculated as follows:

(20) $$\mathrm{Pr}\left\{\Omega_{P}|S=Ø\right\}=\mathrm{Pr}\left\{\log_{2}\left(1+P_{P}{\left|h_{P}\right|}^{2}\right)<R_{P}\right\}=1-e^{-\frac{\rho_{P}N_{0}}{P_{P}\sigma_{P}^{2}}}$$

and

(21) $$\begin{array}{c} \mathrm{Pr}\left\{S=S_{l}\right\}=\prod_{i\in {\bar{S}}_{l}}\mathrm{Pr}\left\{C_{P}<R_{P}\right\}\prod_{j\in S_{l}}\mathrm{Pr}\left\{C_{P}\geq R_{P}\right\}\\ =\prod_{i\in {\bar{S}}_{l}}\left(1-\frac{\sigma_{P}^{2}P_{P}}{\sigma_{P}^{2}P_{P}+\rho_{P}P_{S}\sigma_{S_{i}P}^{2}}e^{-\frac{\rho_{P}N_{0}}{P_{P}\sigma_{P}^{2}}}\right)\prod_{j\in S_{l}}\frac{\sigma_{P}^{2}P_{P}}{\sigma_{P}^{2}P_{P}+\rho_{P}P_{S}\sigma_{S_{i}P}^{2}}e^{-\frac{\rho_{P}N_{0}}{P_{P}\sigma_{P}^{2}}}, \end{array}$$

where $\rho_{P}=2^{R_{P}}-1$. According to the definition of the set $S_{l}$, we can see that $\mathrm{Pr}\{\Omega_{P}|S=S_{l}\}$ equals to zero in OSTS and OCJS. Thus, the expression of the outage probability for the primary system is obtained by substituting Equations (19)–(21) and $\mathrm{Pr}\{\Omega_{P}|S=S_{l}\}=0$ into Equation (17).

### 3.2. The Outage Probability of the Secondary System

We use $\Omega_{S}$ to denote an event, which represents an occurrence of outage of the channel ST→SR. If $C_{S}^{S=S_{l}}<R_{S}$, then the event $\Omega_{S}$ occurs. Therefore, we have

(22) $$S_{\mathrm{out}}=\mathrm{Pr}\left\{S=Ø\right\}\mathrm{Pr}\left\{\Omega_{S}|S=Ø\right\}+\sum_{l=1}^{2^{K-1}-1}\mathrm{Pr}\left\{S=S_{l}\right\}\mathrm{Pr}\left\{\Omega_{S}|S=S_{l}\right\}$$

and

(23) $$\begin{array}{cc} \mathrm{Pr}\left\{\Omega_{S}|S=S_{l}\right\} & =\min_{{\mathrm{ST}}_{i}\in S_{l}}\mathrm{Pr}\left\{C_{S}^{S=S_{l}}<R_{S}\right\}\\ & =\mathrm{Pr}\left\{\max_{{\mathrm{ST}}_{i}\in S_{l}}\frac{P_{S}{\left|h_{S_{i}}\right|}^{2}}{P_{P}{\left|h_{\mathrm{PS}}\right|}^{2}+N_{0}}<2^{R_{S}}-1\right\}\\ & =\prod_{i=1}^{L-1}\mathrm{Pr}\left\{\frac{P_{S}{\left|h_{S_{i}}\right|}^{2}}{P_{P}{\left|h_{\mathrm{PS}}\right|}^{2}+N_{0}}<2^{R_{S}}-1\right\}. \end{array}$$

Furthermore, $|h_{S_{i}}{|}^{2}$ and $|h_{\mathrm{PS}}{|}^{2}$ are i.i.d. exponential distribution with parameters $1/\sigma_{S_{i}}^{2}$ and $1/\sigma_{\mathrm{PS}}^{2}$, respectively. Let $Z_{1}={\left|h_{S_{i}}\right|}^{2}$ and $Z_{2}={\left|h_{\mathrm{PS}}\right|}^{2}$, thus Equation (23) can be rewritten as

(24) $$\begin{array}{cc} \mathrm{Pr}\left\{\Omega_{S}|S=S_{l}\right\} & =\prod_{i=1}^{L-1}\mathrm{Pr}\left\{\frac{P_{S}Z_{1}}{P_{P}Z_{2}+N_{0}}<\rho_{S}\right\}\\ & =\prod_{i=1}^{L-1}\int_{0}^{\infty }\frac{1}{\sigma_{{\mathrm{PS}}_{i}}^{2}}e^{-\frac{z_{2}}{\sigma_{{\mathrm{PS}}_{i}}^{2}}}\int_{0}^{\rho_{S}\left(P_{P}z_{1}+N_{0}\right)/P_{S}}\frac{1}{\sigma_{S_{i}}^{2}}e^{-\frac{z_{1}}{\sigma_{S_{i}}^{2}}}dz_{1}dz_{2}\\ & =\prod_{i=1}^{L-1}\left(1-\frac{\sigma_{S_{i}}^{2}P_{S}e^{-\frac{\rho_{S}N_{0}}{P_{S}\sigma_{S_{i}}^{2}}}}{\sigma_{S_{i}}^{2}P_{S}+\rho_{S}P_{P}\sigma_{{\mathrm{PS}}_{i}}^{2}}\right), \end{array}$$

where $\rho_{S}=2^{R_{S}}-1$. We can see that $\mathrm{Pr}\{\Omega_{S}|S=Ø\}=1$. Thus, the expression of the outage probability of the secondary system is obtained by substituting Equations (19), (21), (24) and $\mathrm{Pr}\{\Omega_{S}|S=Ø\}=1$ into Equation (22).

### 3.3. The Intercept Probability of the Primary Transmission

The secrecy intercept event for the primary system is denoted by $\Omega_{\mathrm{int}}$. Hence, the intercept probability of the primary transmission is equal to the probability of the event $\Omega_{\mathrm{int}}$ occurrence [33]. In addition, the secrecy intercept event occurs when $C_{P}^{S=Ø}<C_{E}^{S=Ø}$ or $C_{P}^{S\neq Ø}<C_{E}^{S\neq Ø}$. Obviously, the event $\Omega_{\mathrm{int}}$ occurs only when S≠Ø. Therefore, we obtain

(25) $$P_{\mathrm{int}}=\mathrm{Pr}\left\{S=Ø\right\}\mathrm{Pr}\left\{\Omega_{\mathrm{int}}|S=Ø\right\}+\sum_{l=1}^{2^{K-1}-1}\mathrm{Pr}\left\{S=S_{l}\right\}\mathrm{Pr}\left\{\Omega_{\mathrm{int}}|S=S_{l}\right\}.$$

Moreover, $|h_{\mathrm{PE}}{|}^{2}$, $|h_{S_{i}E}{|}^{2}$ and $|h_{S_{o}E}{|}^{2}$ are exponential variables with parameters $1/\sigma_{\mathrm{PE}}^{2}$, $1/\sigma_{S_{i}E}^{2}$ and $1/\sigma_{S_{o}E}^{2}$, respectively. Let ${\hat{X}}_{3}={\left|h_{S_{i}E}\right|}^{2}$, $X_{3}={\left|h_{S_{o}E}\right|}^{2}$ and $X_{4}={\left|h_{\mathrm{PE}}\right|}^{2}$. Thus, when S=Ø, the conditional intercept probability $\mathrm{Pr}\{\Omega_{\mathrm{int}}|S=Ø\}$ and $\mathrm{Pr}\{\Omega_{\mathrm{int}}|S=S_{l}\}$ can be derived as

(26) $$\begin{array}{cc} \mathrm{Pr}\{\Omega_{\mathrm{int}}|S=Ø\} & =\mathrm{Pr}\left\{\frac{P_{P}{\left|h_{P}\right|}^{2}}{N_{0}}<\frac{P_{P}{\left|h_{\mathrm{PE}}\right|}^{2}}{P_{S}\cdot \max_{o\in \{1,\ldots ,K\}}{\left|h_{S_{o}E}\right|}^{2}+N_{0}}\right\}\\ & =\prod_{o\in \{1,\ldots ,K\}}\mathrm{Pr}\left\{|h_{P}{|}^{2}<\frac{N_{0}}{P_{S}}\frac{|h_{\mathrm{PE}}{|}^{2}}{|h_{S_{o}E}{|}^{2}+N_{0}/P_{S}}\right\}\\ & =\prod_{o\in \{1,\ldots ,K\}}\mathrm{Pr}\left\{X_{1}<\frac{N_{0}}{P_{S}}\frac{X_{4}}{X_{3}+N_{0}/P_{S}}\right\} \end{array}$$

and

(27) $$\begin{array}{cc} \mathrm{Pr}\{\Omega_{\mathrm{int}}|S=S_{l}\} & =\min_{o\in \{1,\ldots ,K\}}\mathrm{Pr}\left\{C_{P}^{S=S_{l}}<C_{E}^{S=S_{l}}\right\}\\ & =\mathrm{Pr}\left\{C_{P}^{S=S_{l}}<\min_{o\in \{1,\ldots ,K\}}C_{E}^{S=S_{l}}\right\}\\ & =\prod_{o=1}^{K}\mathrm{Pr}\left\{C_{P}^{S=S_{l}}<C_{E}^{S=S_{l}}\right\}\\ & =\prod_{o=1}^{K}\mathrm{Pr}\left\{\frac{P_{P}{\left|h_{P}\right|}^{2}}{P_{S}{\left|h_{S_{i}P}\right|}^{2}+N_{0}}<\frac{P_{P}{\left|h_{\mathrm{PE}}\right|}^{2}}{P_{S}|h_{S_{i}E}{|}^{2}+P_{S}{\left|h_{S_{o}E}\right|}^{2}+N_{0}}\right\}\\ & =\prod_{o=1}^{K}\mathrm{Pr}\left\{\frac{X_{1}}{X_{2}+N_{0}/P_{S}}<\frac{X_{4}}{X_{3}+{\hat{X}}_{3}+N_{0}/P_{S}}\right\}. \end{array}$$

Let ${\tilde{X}}_{2}=X_{2}+N_{0}/P_{S}$, ${\tilde{X}}_{3}=X_{3}+{\hat{X}}_{3}+N_{0}/P_{S}$, $Y_{1}=X_{1}/{\tilde{X}}_{2}$, $Y_{2}=X_{4}/{\tilde{X}}_{3}$, and $Y_{3}=X_{4}/\left(X_{3}+N_{0}/P_{S}\right)$. Following Equations (A1) and (A9) in Appendix A, the probability density of random variables $Y_{1}$, $Y_{2}$ and $Y_{3}$ can be written as follows:

(28) $$\begin{array}{cc} f_{Y_{1}}\left(y_{1}\right) & =\left(\frac{\sigma_{P}^{2}/\sigma_{S_{i}P}^{2}}{{\left(\sigma_{P}^{2}/\sigma_{S_{i}P}^{2}+y_{1}\right)}^{2}}+\frac{N_{0}/\left(P_{S}\sigma_{S_{i}P}^{2}\right)}{\sigma_{P}^{2}/\sigma_{S_{i}P}^{2}+y_{1}}\right)e^{-\frac{N_{0}y_{1}}{P_{S}\sigma_{P}^{2}}}\\ & =\left(\frac{a_{1}}{{\left(a_{1}+y_{1}\right)}^{2}}+\frac{a_{1}b_{1}}{a_{1}+b_{1}}\right)e^{-b_{1}y_{1}}, \end{array}$$

(29) $$\begin{array}{cc} f_{Y_{2}}\left(y_{2}\right) & =\left(\frac{1}{{\left(\sigma_{\mathrm{PE}}^{2}/\sigma_{S_{o}E}^{2}+y_{2}\right)}^{2}}+\frac{N_{0}/\left(P_{S}\sigma_{\mathrm{PE}}^{2}\right)}{\sigma_{\mathrm{PE}}^{2}/\sigma_{S_{o}E}^{2}+y_{2}}\right)\cdot \frac{\sigma_{\mathrm{PE}}^{2}e^{-\frac{N_{0}y_{2}}{P_{S}\sigma_{\mathrm{PE}}^{2}}}}{\sigma_{S_{o}E}^{2}-\sigma_{S_{i}E}^{2}}\\ & =c\left(\frac{1}{{\left(a_{2}+y_{2}\right)}^{2}}+\frac{b_{2}}{a_{2}+y_{2}}\right)e^{-b_{2}y_{2}}, \end{array}$$

and

(30) $$\begin{array}{cc} f_{Y_{3}}\left(y_{3}\right) & =\left(\frac{\sigma_{\mathrm{PE}}^{2}/\sigma_{S_{o}E}^{2}}{{\left(\sigma_{\mathrm{PE}}^{2}/\sigma_{S_{o}E}^{2}+y_{3}\right)}^{2}}+\frac{N_{0}/\left(P_{S}\sigma_{S_{o}E}^{2}\right)}{\sigma_{\mathrm{PE}}^{2}/\sigma_{S_{o}E}^{2}+y_{3}}\right)e^{-\frac{N_{0}y_{3}}{P_{S}\sigma_{\mathrm{PE}}^{2}}}\\ & =\left(\frac{a_{3}}{{\left(a_{3}+y_{3}\right)}^{2}}+\frac{a_{3}b_{3}}{a_{3}+b_{3}}\right)e^{-b_{3}y_{3}}, \end{array}$$

where $a_{1}=\sigma_{P}^{2}/\sigma_{S_{i}P}^{2}$, $b_{1}=N_{0}/P_{S}\sigma_{P}^{2}$, $a_{2}=a_{3}=\sigma_{\mathrm{PE}}^{2}/\sigma_{S_{o}E}^{2}$, $b_{2}=b_{3}=N_{0}/P_{S}\sigma_{\mathrm{PE}}^{2}$, $c=\sigma_{S_{o}E}^{2}/\left(\sigma_{S_{o}E}^{2}-\sigma_{S_{i}E}^{2}\right)$. By using the equalities in Equations (28)–(30), Equations (26) and (27) can be rewritten, respectively, as follows:

(31) $$\begin{array}{c} \mathrm{Pr}\left\{\Omega_{\mathrm{int}}|S=Ø\right\}=\prod_{o\in \left\{1,\ldots ,K\right\}}\mathrm{Pr}\left\{X_{1}<\left(N_{0}/P_{S}\right)Y_{3}\right\}\\ =\prod_{o\in \left\{1,\ldots ,K\right\}}\int_{0}^{\infty }\left(\frac{a_{3}}{{\left(a_{3}+y_{3}\right)}^{2}}+\frac{a_{3}b_{3}}{a_{3}+b_{3}}\right)e^{-b_{3}y_{3}}\int_{0}^{\frac{N_{0}}{P_{S}}}\frac{1}{\sigma_{P}^{2}}e^{-\frac{x_{1}}{\sigma_{P}^{2}}}dx_{1}dy_{3}\\ =-\prod_{o\in \left\{1,\ldots ,K\right\}}a_{3}b_{1}e^{a_{3}\left(b_{1}+b_{3}\right)}\mathrm{Ei}\left(-a_{3}\left(b_{1}+b_{3}\right)\right) \end{array}$$

and

(32) PrΩint|S=Sl=∏o=1KPrY1<Y2=∏o=1K∫0∞c+cb2y2+a2y2+a22e−b2y2∫0y2a1+a1b1y1+a1y1+a12e−b1y1dy1dy2=∏o=1Kc−a1a2c1+a1b1−a2b1a1−a22ea2b1+b2Ei−a2b1+b2a1a2c1+a2b2−a1b2a1−a22a1a2c1+a2b2−a1b2a1−a22+a1a2c1+a2b2−a1b2a1−a22ea1b1+b2Ei−a1b1+b2−a1ca1−a2,

where $\mathrm{Ei}\left(x\right)=\int_{-\infty }^{x}\frac{1}{x}e^{x}dx=r+\ln\left(-x\right)+\sum_{k=1}^{\infty }\frac{x^{k}}{k\cdot k!}$, x<0, *r* is the Euler’s constant. Therefore, the intercept probability of the primary system in proposed protocol is obtained by substituting Equations (19), (21), (31) and (32) into Equation (25).

### 3.4. The Outage and Intercept Probability for the Conventional No-Security Protocol

Similar to the performance analysis for the proposed protocols, the primary and secondary outage probability and the primary intercept probability are calculated, respectively, as follows:

(33) $$P_{\mathrm{out}}^{C}=\mathrm{Pr}\left\{S^{C}=Ø\right\}\mathrm{Pr}\left\{\Omega_{P}|S^{C}=Ø\right\}+\sum_{l=1}^{2^{K}-1}\mathrm{Pr}\left\{S^{C}=S_{l}\right\}\mathrm{Pr}\left\{\Omega_{P}|S^{C}=S_{l}\right\},$$

(34) $$S_{\mathrm{out}}^{C}=\mathrm{Pr}\left\{S^{C}=Ø\right\}\mathrm{Pr}\left\{\Omega_{S}|S^{C}=Ø\right\}+\sum_{l=1}^{2^{K}-1}\mathrm{Pr}\left\{S^{C}=S_{l}\right\}\mathrm{Pr}\left\{\Omega_{S}|S^{C}=S_{l}\right\},$$

and

(35) $$P_{\mathrm{int}}^{C}=\mathrm{Pr}\left\{S^{C}=Ø\right\}\mathrm{Pr}\left\{\Omega_{\mathrm{int}}|S^{C}=Ø\right\}+\sum_{l=1}^{2^{K}-1}\mathrm{Pr}\left\{S^{C}=S_{l}\right\}\mathrm{Pr}\left\{\Omega_{\mathrm{int}}|S^{C}=S_{l}\right\}.$$

We can see that $\mathrm{Pr}\{\Omega_{S}|S^{C}=Ø\}=1$, $\mathrm{Pr}\{\Omega_{P}|S^{C}=S_{l}\}=1$. To encourage STs to aid the transmission of artificial noise, we set $R_{P_{0}}\geq R_{P}$. Therefore, we also have

(36) $$\mathrm{Pr}\left\{S^{C}=Ø\right\}=\prod_{i=1}^{K}\mathrm{Pr}\left\{C_{P}^{C}<R_{P_{0}}\right\}=\prod_{i=1}^{K}\left(1-\frac{\sigma_{P}^{2}P_{P}}{\sigma_{P}^{2}P_{P}+\rho_{P_{0}}P_{S}\sigma_{S_{i}P}^{2}}e^{-\frac{\rho_{P_{0}}N_{0}}{P_{P}\sigma_{P}^{2}}}\right),$$

(37) $$\begin{array}{cc} \mathrm{Pr}\left\{S^{C}=S_{l}\right\} & =\prod_{i\in {\bar{S}}_{l}}\mathrm{Pr}\left\{C_{P}^{C}<R_{P_{0}}\right\}\prod_{j\in S_{l}}\mathrm{Pr}\left\{C_{P}^{C}\geq R_{P_{0}}\right\}\\ & =\prod_{i\in {\bar{S}}_{l}}\left(1-\frac{\sigma_{P}^{2}P_{P}}{\sigma_{P}^{2}P_{P}+\beta \rho_{P_{0}}P_{S}\sigma_{S_{i}P}^{2}}e^{-\frac{\rho_{P_{0}}N_{0}}{P_{P}\sigma_{P}^{2}}}\right)\\ & \times \prod_{j\in S_{l}}\left(1-\frac{\sigma_{P}^{2}P_{P}}{\sigma_{P}^{2}P_{P}+\beta \rho_{P_{0}}P_{S}\sigma_{S_{j}P}^{2}}e^{-\frac{\rho_{P_{0}}N_{0}}{P_{P}\sigma_{P}^{2}}}\right), \end{array}$$

(38) $$\mathrm{Pr}\left\{\Omega_{P}|S^{C}=Ø\right\}=\mathrm{Pr}\left\{\log_{2}\left(1+P_{P}{\left|h_{P}\right|}^{2}/N_{0}\right)<R_{P_{0}}\right\}=1-e^{-\frac{\rho_{P_{0}}N_{0}}{P_{P}\sigma_{P}^{2}}},$$

(39) $$\mathrm{Pr}\left\{\Omega_{S}|S^{C}=S_{l}\right\}=\prod_{i=1}^{L}\mathrm{Pr}\left\{C_{S}^{C}<R_{S}\right\}=\prod_{i=1}^{L}\left(1-\frac{\sigma_{S_{i}}^{2}P_{S}}{\sigma_{S_{i}}^{2}P_{P}+\rho_{S}P_{P}\sigma_{{\mathrm{PS}}_{i}}^{2}}e^{-\frac{\rho_{S}N_{0}}{P_{S}\sigma_{S_{i}}^{2}}}\right),$$

(40) $$\begin{array}{cc} \mathrm{Pr}\left\{\Omega_{\mathrm{int}}|S^{C}=Ø\right\} & =\mathrm{Pr}\left\{P_{P}|h_{P}{|}^{2}/N_{0}<P_{P}{\left|h_{\mathrm{PE}}\right|}^{2}/N_{0}\right\}=\mathrm{Pr}\left\{X_{1}<X_{4}\right\}\\ & =\int_{0}^{\infty }\frac{1}{\sigma_{\mathrm{PE}}^{2}}e^{-\frac{x_{4}}{\sigma_{\mathrm{PE}}^{2}}}\int_{0}^{x_{4}}\frac{1}{\sigma_{P}^{2}}e^{-\frac{x_{1}}{\sigma_{P}^{2}}}dx_{1}dx_{4}\\ & =1-\frac{\sigma_{P}^{2}}{\sigma_{P}^{2}+\sigma_{\mathrm{PE}}^{2}}, \end{array}$$

(41) $$\begin{array}{cc} \mathrm{Pr}\left\{\Omega_{\mathrm{int}}|S^{C}=S_{l}\right\} & =\mathrm{Pr}\left\{C_{P}^{C}<C_{E}^{C}\right\}\\ & =\mathrm{Pr}\left\{\frac{P_{P}{\left|h_{P}\right|}^{2}}{P_{S}{\left|h_{S_{o}P}\right|}^{2}+N_{0}}<\frac{P_{\mathrm{PE}}{\left|h_{P}\right|}^{2}}{P_{S}{\left|h_{S_{o}E}\right|}^{2}+N_{0}}\right\}\\ & =1-\frac{a_{1}}{a_{1}-{\tilde{a}}_{2}}+c_{1}\mathrm{Ei}\left(-a_{1}\left(b_{1}+b_{2}\right)\right)\\ & +c_{2}\mathrm{Ei}\left(-{\tilde{a}}_{2}\left(b_{1}+b_{2}\right)\right)e^{{\tilde{a}}_{2}\left(b_{1}+b_{2}\right)}, \end{array}$$

where $\rho_{P_{0}}=2^{R_{P_{0}}}-1$, ${\tilde{a}}_{2}=\sigma_{\mathrm{PE}}^{2}/\sigma_{S_{i}E}^{2}$, $c_{1}=\beta a_{1}{\tilde{a}}_{2}\left(1+{\tilde{a}}_{2}b_{2}-\beta a_{1}b_{2}\right)/{\left({\tilde{a}}_{2}-\beta a_{1}\right)}^{2}$ and $c_{2}=\beta a_{1}{\tilde{a}}_{2}\left({\tilde{a}}_{2}b_{1}-\beta a_{1}b_{1}-1\right)/{\left({\tilde{a}}_{2}-\beta a_{1}\right)}^{2}$.

## 4. Numerical Results

The simulation results of the proposed protocols are provided in this section. The systems comprise a primary pair (PS-PD), an eavesdropper (E), a secondary receiver (SR) and *K* secondary transmitters ${\mathrm{ST}}_{i}$ (i=1,…,K). Since the secondary user can serve as cooperative jammer, the primary user relaxes the interference threshold in return, which decreases the minimum achievable rate of primary user $R_{P}$. Thus, we set $R_{P}=1.5$ Bit/s/Hz and $R_{P}=1$ Bit/s/Hz in the conventional model and the proposed model, respectively. If the parameters are not specified, the simulation parameters are settled as follow: $R_{S}=1$ Bit/s/Hz; $r_{1}=10\mathrm{lg}\left(P_{P}/N_{0}\right)=10$ dB is the average transmit SNR of the primary user. In addition, $\sigma_{P}^{2}=\sigma_{\mathrm{SP}}^{2}=\sigma_{\mathrm{PS}}^{2}=\sigma_{\mathrm{PE}}^{2}=1$, $\sigma_{S_{o}E}^{2}=3$, $\sigma_{S_{i}E}^{2}=1/5$ and $\sigma_{S}^{2}=4$.

**Figure 2:**
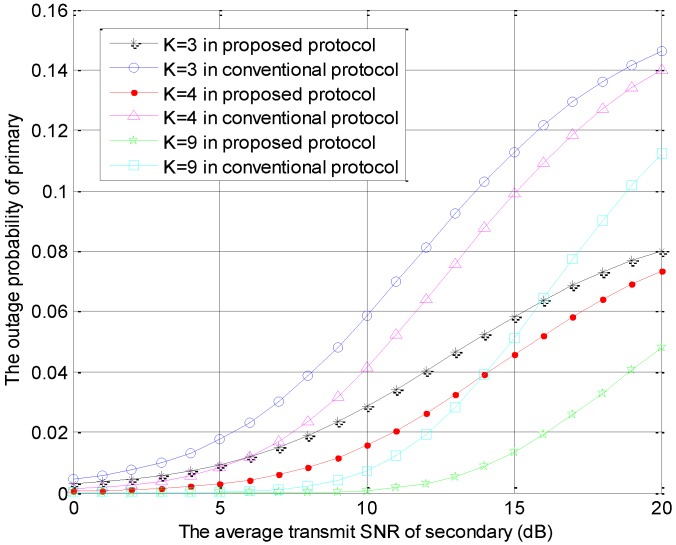
The outage probabilities of primary users versus $r_{2}$ with different *K* values.

The outage probabilities of the primary user versus $r_{2}$ in the conventional model and the proposed model are shown as Figure 2, where $r_{2}=10\mathrm{lg}\left(P_{S}/N_{0}\right)$. The special parameter is the number of STs, which is fixed as K=3;4;9. In Figure 2, the outage probability of primary system increases with increase of the secondary SNR. In the same protocol, the primary outage probability decreases with increase of the number of secondary users. This is because the diversity gain increases with increase of the number of secondary users. Furthermore, the outage probability of primary system in our proposed protocol is less than the conventional protocol, which is because that the secondary user is encouraged to serve as friendly jammer, which decreases the interference threshold.

**Figure 3:**
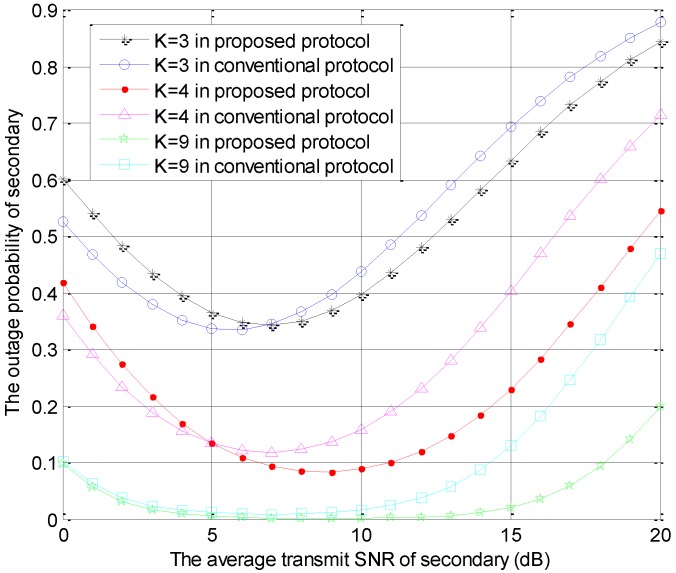
The outage probability of secondary system versus $r_{2}$ in the two protocols with different *K* values.

The outage probabilities of the secondary user versus $r_{2}$ are shown in Figure 3, which is generated by using the same parameters as those in Figure 2. In Figure 3, in the same protocol, the secondary outage performances are improved when the number of STs becomes larger. Moreover, the outage probabilities of secondary decrease firstly, and increase with the increase of the average SNR for secondary in the two protocols. Furthermore, the increasing trend is due to that the interference threshold is always not satisfied by secondary user when the SNR of the secondary user is too large. In the small secondary average SNR range, the outage performance of secondary users in the conventional model is better than the performance in the proposed protocol. This performance is mainly determined by the multi-user diversity gain. In this case, the proposed protocol has a lower multi-user diversity gain than the conventional model due to one secondary transmitter acting as the cooperative jammer. In contrast, the proposed protocol can provide a better secondary outage performance in the high secondary average SNR range because the primary user relaxes the interference threshold.

**Figure 4:**
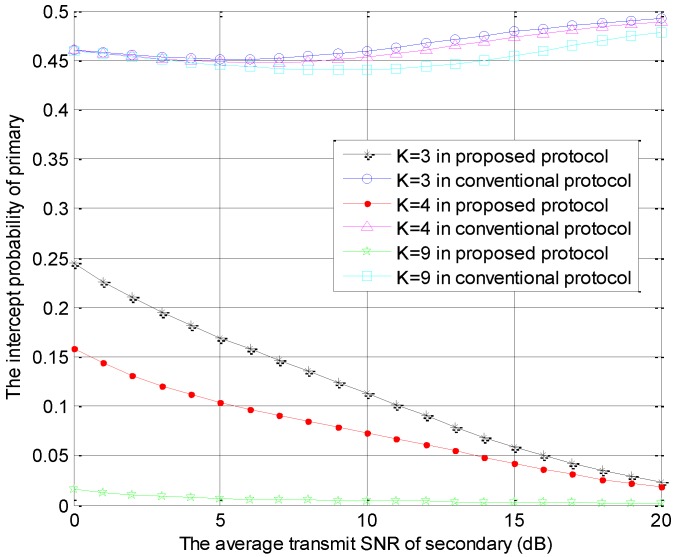
The intercept probabilities of primary users versus $r_{2}$ with different *K* values.

**Figure 5:**
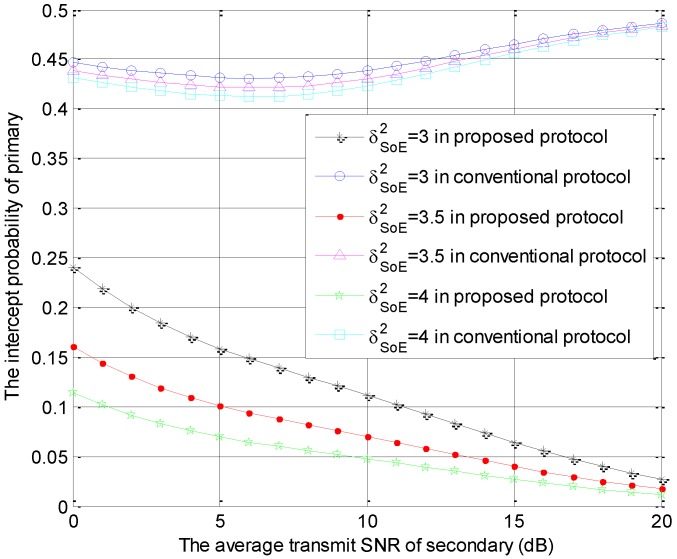
The intercept probabilities of primary users versus $r_{2}$ with different $\sigma_{\mathrm{SE}}^{2}$ values.

**Figure 6:**
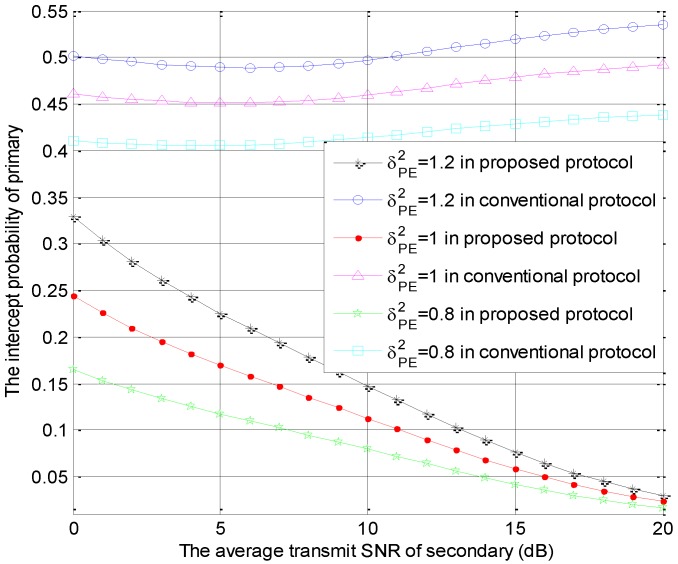
The intercept probabilities of primary users versus $r_{2}$ with different $\sigma_{\mathrm{PE}}^{2}$ values.

**Figure 7:**
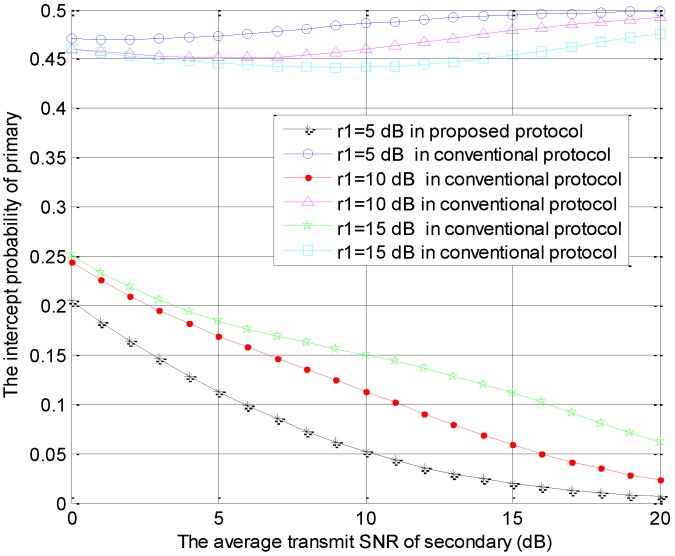
The intercept probabilities of primary users versus $r_{2}$ with different $r_{1}$ values.

Figure 4 is generated using the same parameters as those in Figure 2, which shows the intercept probabilities of the primary versus $r_{2}$ with different number of STs. In Figure 4, the primary security performance is improved significantly in the proposed protocol and is improved slightly in the conventional model as the number of STs becomes larger due to the multi-user diversity gain. Moreover, compared with the conventional protocol, our protocol can provide better primary security performance. The intercept probabilities of the primary system decrease with the increase of $r_{2}$ in the proposed protocol because the interference from ST to eavesdropper increases with the increase of $r_{2}$. However, the intercept probabilities of the primary system decrease firstly and increase with the increase of $r_{2}$ in the conventional protocol. In the small value range of $r_{2}$, the interference threshold is always satisfied, but the interference from ST to eavesdropper increases with the increase of $r_{2}$, which causes the decreasing phenomenon. In the large value range of $r_{2}$, the interference threshold is hard to satisfy. Thus, the access probability of the secondary transmission decreases and the interference from ST to eavesdropper is reduced with the increase of $r_{2}$. This is the cause of the latter increasing phenomenon. These numerical results can also be found in Figure 5, Figure 6 and Figure 7.

The intercept probabilities of primary users versus $r_{2}$ with different values of $\sigma_{\mathrm{SE}}^{2}$ are shown in Figure 5. Namely, the special parameter is the channel coefficient $\sigma_{\mathrm{SE}}^{2}$, which equals 3, 3.5 or 4. As described in Figure 5, the primary security performance is improved significantly in the proposed protocol and is improved slightly in the conventional model as the value of $\sigma_{\mathrm{SE}}^{2}$ becomes larger. Compared with the conventional protocol, our protocol can provide the better primary security performance because the larger value of $\sigma_{S_{o}E}^{2}$ represents the better channel conditions for links ${\mathrm{ST}}_{o}\to E$. In other words, the interference from ${\mathrm{ST}}_{o}$ to eavesdropper increases with the increase of $\sigma_{S_{o}E}^{2}$. In addition, the interference to eavesdropper from ${\mathrm{ST}}_{o}$ is greater than that from ${\mathrm{ST}}_{i}$. In proposed protocol, both ${\mathrm{ST}}_{o}$ and ${\mathrm{ST}}_{i}$ interfere with the eavesdropping. However, the interference to eavesdropper just comes from ${\mathrm{ST}}_{i}$ in the conventional model and the probability that *i* is equal to *o* is 1/K.

The intercept probabilities of primary users versus $r_{2}$ with different values of $\sigma_{\mathrm{PE}}^{2}$ as shown in Figure 6. Namely, the special parameter is the channel coefficient $\sigma_{\mathrm{PE}}^{2}$, which equals to 1.2, 1 or 0.8. In Figure 6, a smaller value of $\sigma_{\mathrm{PE}}^{2}$ can lead to a good primary security performance in the same protocol because a larger value of $\sigma_{\mathrm{PE}}^{2}$ represents the better channel conditions for links PS→E. In other words, the instantaneous capacity of PS→E increases with the increase of $\sigma_{\mathrm{PE}}^{2}$. In the proposed protocol with the larger values of $\sigma_{\mathrm{PE}}^{2}$, the primary security performance is enhanced significantly in small value range of $r_{2}$ and is enhanced slightly in large value range of $r_{2}$. Compared to the conventional protocol, the proposed protocol can provide the better primary security performance. These numerical results are consistent with those in Figure 4 and Figure 5.

The intercept probabilities of primary users versus $r_{2}$ with different values of $r_{1}$ are shown in Figure 7. Namely, the special parameter is the average SNR of the primary user, which is set as $r_{1}=10\mathrm{lg}\left(P_{P}/N_{0}\right)=5$, 10 or 15 dB. In Figure 7, the primary security performance in the proposed protocol is improved as the value of $r_{1}$ becomes larger. On the contrary, the primary security performance in the conventional model is reduced as the value of $r_{1}$ becomes larger. The valid primary information received by eavesdropper and the interference to eavesdropper are the two main factors related to the security performance of primary system. The more valid primary information is received by the eavesdropper, the worse is primary security performance achieved, and the more interference to he eavesdropper, the greater is primary security performance achieved. In the proposed protocol, the smaller value of $r_{1}$ causes the less valid primary information received at eavesdropper, so the smaller intercept probability of the primary system is obtained. In the conventional model, the interference threshold is hard to satisfy with the smaller $r_{1}$ and the interference caused by ${\mathrm{ST}}_{i}$ to eavesdropper is very little, so the larger intercept probability of the primary system is obtained. All of the above numerical results are consistent with the theoretical results in Section 3.

## 5. Conclusions

In this paper, we have investigated the physical-layer security for a cognitive Internet of things model, which is composed of a primary pair (PS-PD), a secondary receiver (SR), *K* secondary transmitters and an eavesdropper. To protect the information of primary users against eavesdropping, we have proposed the ST cooperative jammer selection transmission protocol. In return, for the cooperation of ${\mathrm{ST}}_{o}$, interference threshold for secondary user is relaxed by the primary system compared with the non-security management model. When this interference threshold is satisfied and the best outage performance of secondary users is obtained by selecting ${\mathrm{ST}}_{i}$, then the secondary user ${\mathrm{ST}}_{i}$ has access to the licensed spectrum. Due to the cooperation of ${\mathrm{ST}}_{o}$, the security performance of primary users are enhanced. Due to the cooperation of ${\mathrm{ST}}_{o}$ and the selection of ${\mathrm{ST}}_{i}$, the outage performance of secondary users are enhanced in high secondary transmit SNR region. Furthermore, the intercept probability and outage probability of the primary system have been derived. The outage probability of the secondary system has also been obtained. For comparison purposes, the conventional non-security management was also investigated as a baseline. The numerical results have shown that our protocol has better primary secrecy performance than the non-security management model. In addition, the proposed protocol also has better secondary and primary transmission performance than the conventional model.

## Appendix A

Let $X_{1}$, $X_{2}$, ${\hat{X}}_{3}$ and $X_{4}$ be exponentially variables with parameters $1/\sigma_{P}^{2}$, $1/\sigma_{S_{i}P}^{2}$, $1/\sigma_{S_{o}E}^{2}$, $1/\sigma_{S_{i}E}^{2}$ and $1/\sigma_{\mathrm{PE}}^{2}$, respectively. Let ${\tilde{X}}_{2}=X_{2}+N_{0}/P_{S}$, ${\tilde{X}}_{3}=X_{3}+{\hat{X}}_{3}+N_{0}/P_{S}$, $Y_{1}=X_{1}/{\tilde{X}}_{2}$, $Y_{2}=X_{4}/{\tilde{X}}_{3}$, and $Y_{3}=X_{4}/\left(X_{3}+N_{0}/P_{S}\right)$. Since

(A1) $$\begin{array}{cc} F_{{\tilde{X}}_{2}}\left({\tilde{x}}_{2}\right) & =\mathrm{Pr}\left\{{\tilde{X}}_{2}<{\tilde{x}}_{2}\right\}=\mathrm{Pr}\left\{X_{2}<{\tilde{x}}_{2}-N_{0}/P_{S}\right\}\\ & =\int_{0}^{{\tilde{x}}_{2}-N_{0}/P_{S}}\frac{1}{\sigma_{S_{i}P}^{2}}e^{-\frac{x_{2}}{\sigma_{S_{i}P}^{2}}}dx_{2}=1-e^{\frac{N_{0}}{P_{S}\sigma_{S_{i}P}^{2}}-\frac{{\tilde{x}}_{2}}{\sigma_{S_{i}P}^{2}}}, \end{array}$$

we have

(A2) $$f_{{\tilde{X}}_{2}}\left({\tilde{x}}_{2}\right)=\frac{1}{\sigma_{S_{i}P}^{2}}e^{\frac{N_{0}}{P_{S}\sigma_{S_{i}P}^{2}}-\frac{{\tilde{x}}_{2}}{\sigma_{S_{i}P}^{2}}}.$$

From Equation (34), we have

(A3) $$\begin{array}{cc} F_{Y_{1}}\left(y_{1}\right) & =\mathrm{Pr}\left\{X_{1}/{\tilde{X}}_{2}<y_{1}\right\}=\mathrm{Pr}\left\{X_{1}<{\tilde{x}}_{2}y_{1}\right\}\\ & =\int_{N_{0}/P_{S}}^{\infty }\frac{1}{\sigma_{S_{i}P}^{2}}e^{-\frac{N_{0}}{P_{S}\sigma_{S_{i}P}^{2}}}e^{-\frac{{\tilde{x}}_{2}}{\sigma_{S_{i}P}^{2}}}\int_{0}^{{\tilde{x}}_{2}y_{1}}\frac{1}{\sigma_{P}^{2}}e^{-\frac{x_{1}}{\sigma_{P}^{2}}}dx_{1}d{\tilde{x}}_{2}\\ & =1-\frac{\sigma_{P}^{2}}{\sigma_{S_{i}P}^{2}y_{1}+\sigma_{P}^{2}}e^{-\frac{N_{0}}{P_{S}\sigma_{P}^{2}}y_{1}}. \end{array}$$

Therefore, we obtain

(A4) $$f_{Y_{1}}\left(y_{1}\right)=\left(\frac{\sigma_{S_{i}P}^{2}\sigma_{P}^{2}}{{\left(\sigma_{P}^{2}+\sigma_{S_{i}P}^{2}y_{1}\right)}^{2}}+\frac{N_{0}/P_{S}}{\sigma_{P}^{2}+\sigma_{S_{i}P}^{2}y_{1}}\right)e^{-\frac{N_{0}y_{1}}{P_{S}\sigma_{P}^{2}}}.$$

Similar to the derivation of the probability density of $Y_{1}$, the probability density of random variables $Y_{3}$ can obtained by

(A5) $$f_{Y_{3}}\left(y_{3}\right)=\left(\frac{\sigma_{S_{o}E}^{2}\sigma_{\mathrm{PE}}^{2}}{{\left(\sigma_{\mathrm{PE}}^{2}+\sigma_{S_{o}E}^{2}y_{3}\right)}^{2}}+\frac{N_{0}/P_{S}}{\sigma_{\mathrm{PE}}^{2}+\sigma_{S_{o}E}^{2}y_{3}}\right)e^{-\frac{N_{0}y_{3}}{P_{S}\sigma_{\mathrm{PE}}^{2}}}.$$

In addition, since

(A6) $$\begin{array}{cc} F_{{\tilde{X}}_{3}}\left({\tilde{x}}_{3}\right) & =\mathrm{Pr}\left\{X_{3}<{\tilde{x}}_{3}-{\hat{x}}_{3}-N_{0}/P_{S}\right\}\\ & =\int_{0}^{\infty }\frac{1}{\sigma_{S_{i}E}^{2}}e^{-\frac{{\hat{x}}_{3}}{\sigma_{S_{i}E}^{2}}}\int_{0}^{{\tilde{x}}_{3}-{\hat{x}}_{3}-N_{0}/P_{S}}\frac{1}{\sigma_{S_{o}P}^{2}}e^{-\frac{x_{3}}{\sigma_{S_{o}P}^{2}}}dx_{3}d{\hat{x}}_{3}\\ & =1-\frac{\sigma_{S_{o}E}^{2}}{\sigma_{S_{o}E}^{2}-\sigma_{S_{i}E}^{2}}e^{\frac{N_{0}}{P_{S}\sigma_{S_{o}E}^{2}}-\frac{{\tilde{x}}_{3}}{\sigma_{S_{o}E}^{2}}}, \end{array}$$

we have

(A7) $$f_{{\tilde{X}}_{3}}\left({\tilde{x}}_{3}\right)=\frac{1}{\sigma_{S_{o}E}^{2}-\sigma_{S_{i}E}^{2}}e^{\frac{N_{0}}{P_{S}\sigma_{S_{o}E}^{2}}-\frac{{\tilde{x}}_{3}}{\sigma_{S_{o}E}^{2}}}.$$

From Equation (A7), we have

(A8) $$\begin{array}{cc} F_{Y_{2}}\left(y_{2}\right) & =\mathrm{Pr}\left\{X_{4}/{\tilde{X}}_{3}<y_{2}\right\}=\mathrm{Pr}\left\{X_{4}<{\tilde{x}}_{3}y_{2}\right\}\\ & =\int_{N_{0}/P_{S}}^{\infty }\frac{1}{\sigma_{S_{o}E}^{2}-\sigma_{S_{i}E}^{2}}e^{\frac{N_{0}}{P_{S}\sigma_{S_{o}E}^{2}}}e^{-\frac{{\tilde{x}}_{3}}{\sigma_{S_{o}E}^{2}}}\int_{0}^{{\tilde{x}}_{3}y_{2}}\frac{1}{\sigma_{\mathrm{PE}}^{2}}e^{-\frac{x_{4}}{\sigma_{PE}^{2}}}dx_{4}d{\tilde{x}}_{3}\\ & =\frac{\sigma_{S_{o}E}^{2}}{\sigma_{S_{o}E}^{2}-\sigma_{S_{i}E}^{2}}\left(1-\frac{\sigma_{\mathrm{PE}}^{2}}{\sigma_{S_{o}E}^{2}y_{2}+\sigma_{\mathrm{PE}}^{2}}e^{-\frac{N_{0}}{P_{S}\sigma_{\mathrm{PE}}^{2}}y_{2}}\right). \end{array}$$

Thus, we obtain

(A9) $$f_{Y_{2}}\left(y_{2}\right)=\frac{e^{-\frac{N_{0}y_{2}}{P_{S}\sigma_{\mathrm{PE}}^{2}}}}{\sigma_{S_{o}E}^{2}-\sigma_{S_{i}E}^{2}}\left(\frac{\sigma_{\mathrm{PE}}^{2}}{{\left(\sigma_{\mathrm{PE}}^{2}/\sigma_{S_{o}E}^{2}+y_{2}\right)}^{2}}+\frac{\sigma_{S_{o}E}^{2}N_{0}/P_{S}}{\sigma_{\mathrm{PE}}^{2}/\sigma_{S_{o}E}^{2}+y_{2}}\right).$$

## References

[1] Wu Q., Ding G., Xu Y., Feng S., Du Z., Wang J., Long K. (2014). Cognitive Internet of Things: A New Paradigm Beyond Connection. IEEE Internet Things J..

[2] Song F., Ai Z., Li J., Pau G., Collotta M., You I., Zhang H. (2017). Smart collaborative caching for Information-centric IoT in fog computing. Sensors.

[3] Ai Z., Liu Y., Song F., Zhang H. (2018). A smart collaborative charging algorithm for mobile power distribution in 5G networks. IEEE Access.

[4] Trihinas D., Pallis G., Dikaiakos M. (2018). Low-Cost Adaptive Monitoring Techniques for the Internet of Things. IEEE Trans. Serv. Comput..

[5] Trihinas D., Pallis G., Dikaiakos M. ADMin: Adaptive Monitoring Dissemination for the Internet of Things. Proceedings of the IEEE INFOCOM 2017—IEEE Conference on Computer Communications.

[6] Tata S., Mohamed M., Megahed A. An Optimization Approach for Adaptive Monitoring in IoT Environments. Proceedings of the 2017 IEEE International Conference on Services Computing (SCC).

[7] Lee Y.-T., Hsiao W.-H., Lin Y.-S., Chou S.-C.T. (2017). Privacy-Preserving Data Analytics in Cloud-Based Smart Home with Community Hierarchy. IEEE Trans. Consum. Electron..

[8] Song F., Zhou Y., Wang Y., Zhao T., You I., Zhang H. (2018). Smart Collaborative Distribution for Privacy Enhancement in Moving Target Defense. Inf. Sci..

[9] Ai Z., Zhou Y., Song F. (2017). A Smart Collaborative Routing Protocol for Reliable Data Diffusion in IoT. Sensors.

[10] Uchida N., Takeuchi S., Ishida T., Shibata Y. (2017). Mobile traffic accident prevention system based on chronological changes of wireless signals and sensors. J. Wirel. Netw. Ubiquitous Comput. Dependable Appl..

[11] Kotenko I., Saenko I., Branitskiy A. (2017). Applying Big Data Processing and Machine Learning Methods for Mobile Internet of Things Security Monitoring. J. Internet Serv. Inf. Secur..

[12] Kotenko I., Saenko I., Kushnerevich A. (2018). Parallel big data processing for security monitoring in Internet of Things networks. J. Wirel. Netw. Ubiquitous Comput. Dependable Appl..

[13] Afza A., Zaidi S.A.R., Shakir M.Z., Imran M.A., Ghogho M. (2015). The Cognitive Internet of Things: A Unified Perspective. IEEE Mob. Netw. Appl..

[14] Jackson D., Zang W., Gu Q., Yu M. (2015). Robust detection of rogue signals in cooperative spectrum sensing. J. Internet Serv. Inf. Secur..

[15] Mitola J. (2000). Cognitive Radio: An Integrated Agent Architecture for Software Defined Radio. Ph.D. Thesis.

[16] Haykin S. (2005). Cognitive radio: Brain-empowered wireless communications. IEEE J. Sel. Areas Commun..

[17] Goldsmith A., Jafar S., Maric I., Srinivasa S. (2009). Breaking spectrum gridlock with cognitive radios: An information theoretic perspective. Proc. IEEE.

[18] Rajesh K.S., Danda B.R. (2015). Advances on Security Threats and Countermeasures for Cognitive Radio Networks: A Survey. IEEE Commun. Surv. Tutor..

[19] Li J., Feng Z., Feng Z., Zhang P. (2015). A Survey of Security Issues in Cognitive Radio Networks. IEEE J. Mag. China Commun..

[20] Nguyen V.D., Hoang T.M., Shin O.S. (2015). Secrecy capacity of the primary system in a cognitive radio network. IEEE Trans. Veh. Technol..

[21] Yulong Z., Xianbin W., Weiming S. (2013). Physical-Layer Security with Multiuser Scheduling in Cognitive Radio Networks. IEEE Trans. Commun..

[22] Zhihui S., Yi Q., Song C. (2013). On physical layer security for cognitive radio networks. IEEE Netw..

[23] Wyner A.D. (1975). The wire-tap channel. Bell Syst. Tech. J..

[24] Zhang N., Lu N., Cheng N., Mark J.W., Shen X.S. (2013). Cooperative spectrum access towards secure information transfer for CRNs. IEEE J. Sel. Areas Commun..

[25] Mokari N., Parsaeefard S., Saeedi H., Azmi P. (2014). Cooperative secure resource allocation in cognitive radio networks with guaranteed secrecy rate for primary users. IEEE Trans. Wirel. Commun..

[26] Xu D., Li Q. (2018). Resource allocation for cognitive radio with primary user secrecy outage constraint. IEEE Syst. J..

[27] Wang C., Wang H.-M. (2014). On the secrecy throughput maximization for MISO cognitive radio network in slow fading channels. IEEE Trans. Inf. Forensics Secur..

[28] Nguyen V.-D., Duong T.Q., Dobre O.A., Shin O.-S. (2016). Joint information and jamming beamforming for secrecy rate maximization in cognitive radio networks. IEEE Trans. Inf. Forensics Secur..

[29] Elkashlan M., Wang L., Duong T.Q., Karagiannidis G.K., Nallanathan A. (2015). On the security of cognitive radio networks. IEEE Trans. Veh. Technol..

[30] Yang L., Jiang H., Vorobyov S.A., Chen J., Zhang H. (2016). Secure communications in underlay cognitive radio networks: User scheduling and performance analysis. IEEE Commun. Lett..

[31] Zou Y. (2017). Physical-layer security for spectrum sharing systems. IEEE Trans. Wirel. Commun..

[32] Pei Y., Liang Y.-C., Teh K.C., Li K. (2011). Secure communication in multiantenna cognitive radio networks with imperfect channel state information. IEEE Trans. Signal Process..

[33] Wang Z., Xiao M., Skoglund M., Poor H.V. (2015). Secure degrees of freedom of wireless networks using artificial noise alignment. IEEE Trans. Commun..

